# Blood loss in primary total knee arthroplasty—body temperature is not a significant risk factor—a prospective, consecutive, observational cohort study

**DOI:** 10.1186/s13018-015-0241-5

**Published:** 2015-06-26

**Authors:** Michael Dan, Sara Martinez Martos, Elaine Beller, Peter Jones, Ray Randle, David Liu

**Affiliations:** Orthopaedic Department, John Hunter Hospital, Lookout Road, New Lambton, 2305 New South Wales Australia; Department of Medicine, Bond University, Gold Coast, Queensland Australia; Gold Coast Centre for Bone and Joint Surgery, Gold Coast, Australia; Centre for Research in Evidence-Based Practice, Bond University, Gold Coast, Queensland Australia

**Keywords:** Hypothermia, Temperature, Knee, Arthroplasty, Blood management

## Abstract

**Background:**

Hypothermia related to anaesthesia and operating theatre environment is associated with increased blood loss in a number of surgical disciplines, including total hip arthroplasty. The influence of patient temperature on blood loss in total knee arthroplasty (TKA) has not been previously studied.

**Methods:**

We recorded patient axillary temperature in the peri-operative period, up to 24 h post-operatively, and analysed the effect on transfusion rate and blood loss from a consecutive cohort of 101 patients undergoing primary TKA.

**Results:**

No relationship between peri-operative patient temperature and blood loss was found within the recorded patient temperature range of 34.7–37.8 °C. Multivariable analysis found increasing age, surgical technique, type of anaesthesia and the use of anti-platelet and anticoagulant medications as significant factors affecting blood loss following TKA.

**Conclusion:**

Patient temperature within a clinically observed range does not have a significant impact on blood loss in primary TKA patients. As long as patient temperature is maintained within a reasonable range during the intra-operative and post-operative periods, strategies other than rigid temperature control above 36.5 °C may be more effective in reducing blood loss following TKA.

## Introduction

Blood loss during joint arthroplasty surgery can be significant and substantial, rendering the patient at risk of requiring an allogenic blood transfusion. Recent data has demonstrated arthroplasty and fracture surgery to account for 9.8 % of all transfused red blood cell units. It was the number one reason for transfusion in patients undergoing surgery, second only to haematology and oncology when considering all medical subspecialties [1]. Therefore, every effort should to be made to reduce the potential for blood loss and requiring allogenic blood during total knee arthroplasty (TKA).

Anaesthetic agents impair the control of body temperature and in combination with operating room temperature are largely responsible for the hypothermia associated with surgery. These agents result in loss of thermoregulatory controls through reduction of shivering and vasoconstriction [2–5]. Hypothermia results from an initial redistribution of heat from the core to the periphery, followed by ongoing decreased metabolic heat production and an increased cutaneous skin loss [6]. Surgical patients without active warming measures typically undergo a 1–2 °C fall in body temperature.

As a result, hypothermia during surgery theoretically produces increased blood loss. Hypothermia impairs the clotting cascade, resulting in increased prothrombin and activated partial thromboplastin times [7]. A decreased body temperature also decreases the production of thromboxane A2, which is responsible for platelet aggregation and activation, as well as vasoconstriction. The decrease in thromboxane A2 results in increased bleeding times [8, 9].

Whether hypothermia-induced coagulopathy results in clinically significant increased blood loss remains controversial. A meta-analysis of studies investigating hypothermia in different surgical specialties concluded that mild hypothermia does increase blood loss [10]. Three studies have looked at the effect of hypothermia and blood loss in total hip arthroplasty, with varied conclusions. Schmied et al. [11] and Winkler et al. [12] demonstrated significantly less blood loss in the warmer patient group, with as little as 0.5–1.5 °C temperature difference. In contrast, Johansson et al. [13] found no significant difference between the two groups with an average 0.8 °C variation in temperature. A retrospective study showed active warming reduces transfusion requirements in total hip and knee replacements, but this study failed to demonstrate a change in haemoglobin levels with treatment [14]. Despite conflicting results in the literature, NHMRC guidelines recommend the use of hypothermia prevention strategies to reduce the incidence of transfusion and blood loss during surgery.

In order to clarify the impact of patient temperature on blood loss in TKA, we undertook this study. Our aim was to determine if peri-operative body temperature affects blood loss during TKA. The study hypothesis is strict maintenance of patient temperature above 36.5 °C will lead to a significant reduction in blood loss in patients undergoing primary TKA.

## Methods

The study is a prospective, consecutive, observational cohort of 101 patients undergoing primary TKA between January and June 2013 by two surgeons in the same orthopaedic department. Revision and bilateral procedures were excluded from the study. Ethics approval was obtained through the Hospital’s Regional Ethics Committee, and all patients gave informed consent prior to inclusion in the study.

A medial parapatellar approach without tourniquet was used in all patients. One surgeon (surgeon B) used computer navigation for alignment and preparation, with cementation of all components in 55 patients. The other surgeon (surgeon A) employed intramedullary instrumentation, with a hybrid prosthesis for the remaining 46 patients. The patella was resurfaced in all patients. All knees were closed in layers with an intra-articular drain, which was removed in day 1 post-operatively. All patients commenced immediate weight bearing and active range of motion from day 1 post-operatively.

Both surgeons used mechanical devices for deep vein thrombosis (DVT) prophylaxis, together with either rivaroxaban 10 mg daily initiated 6 h post operation (surgeon A) or enoxaparin 40 mg daily initiated 4 h post operation (surgeon B). Patients already on coumadin pre-operatively or with a history of previous pulmonary emboli continued their coumadin peri-operatively, aiming for a target international normalized ratio (INR) of 2 on the day of surgery. In addition, aspirin (100 mg) was used in patients deemed high risk for cerebrovascular or cardiovascular complications. The patients on clopidogrel converted to aspirin 100 mg daily, after ceasing clopidogrel 5 days pre-operatively.

Patient axillary temperature was recorded at specific time points during the peri-operative period, including pre-operatively in the anaesthetic bay, post-operatively prior to leaving the operating theatre, prior to exiting the recovery room and then at six hourly intervals for the first 24 h post-operatively. The patients were divided into three temperature groups; less than 36 °C, between 36 and 36.5 °C and greater than 36.5 °C.

Blood loss was measured using three methods: (1) intra-operative blood loss, calculated as the difference between total suction volume and known irrigation volume; (2) post-operative blood loss using drain volume; and (3) total blood loss using the difference between pre-operative and day 1 post-operative haemoglobin levels.

Data on other factors believed to affect blood loss was also collected. These included age, sex, body mass index, heart rate, blood pressure, type of anaesthesia, operation time and the use of anticoagulant and anti-platelet medications [15, 16]. The number of units of allogenic blood transfused for each patient was recorded. The transfusion trigger was haemoglobin of less than 70 g/L or less than 100 g/L in patients with symptomatic anaemia or significant comorbidities [17].

Prior to study commencement, a power calculation was performed. To achieve 80 % power with 5 % significance level, 21 patients were required in each temperature group to detect a clinically significant difference of 8 g/L of haemoglobin between the low and normal temperature groups. Given the study was observational, estimation from previous studies on temperature and blood loss in THA required at least 100 enrolled patients to achieve 21 patients in the low temperature group. Results were described using proportions, means and standard deviations. Simple and multivariable linear regression was used to determine predictors of each of the three measures of blood loss. Interaction terms were fitted between significant predictors of the outcomes and the temperature group variable.

## Results

The patient demographics are summarized in Table 1. There is no statistically significant difference between the surgeons in mean age of their patients (68.1 versus 68.5). Surgeon A had 60.1 % male patients, whereas B had 49.9 % male patients.

**Table 1:** Patient demographics, pre- and post-operative findings

Characteristic	Statistic
Total number of patients	101
Gender	
Male	55 (56.5 %)
Female	46 (45.5 %)
Age mean (SD)	68.6 (9.2)
BMI mean (SD)	30.6 (4.8)
Surgical technique	
Surgeon A intramedullary	46 (45.5 %)
Surgeon B computer navigation	55 (56.5 %)
Heart rate mean (SD)	68 (12)
Systolic blood pressure mean (SD)	131 (23)
Anaesthetic	
General	7 (6.9 %)
Spinal	2 (2.0 %)
Regional	3 (3.0 %)
General and regional	38 (37.6 %)
General and spinal	1 (1.0 %)
Spinal and regional	37 (36.6 %)
General spinal and regional	13 (12.9 %)
Operative time mean (SD)	81 min (33)
Anticoagulant/antiplatelet medications	
Rivaroxaban	40 %
Low-molecular-weight Heparin	38 %
Aspirin	8 %
Warfarin	5 %
Other	9 %

**Fig. 1:**
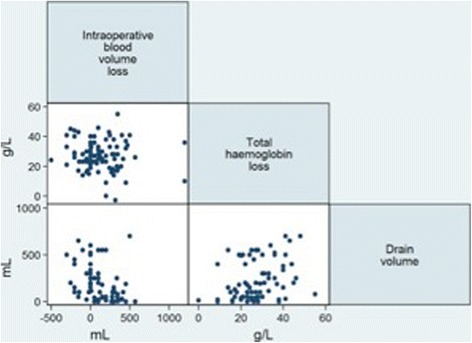
Scatterplot matrix of the three measures of blood loss

The mean operating theatre temperature was 19.6 °C. The average operative time was 81 min (SD 33 min). The mean pre-operative haemoglobin was 143 g/L (SD 14). The mean estimated intra-operative blood loss was 128 mL (SD 266). The average post-operative drain volume was 255 mL (SD 213), with an average haemoglobin difference from pre-operative to day 1 post-operative of 27.2 g/L (SD 10.5). There was no significant correlation between each of the three outcome measures of blood loss, as shown in Fig. 1.

Pre-operatively, patient temperature ranged from 34.3 to 37.2 °C, and post-operatively from 34.7 to 37.8 °C. The minimum temperature recorded at any time point was 34.3 °C. Only two patients recorded temperatures below 35 °C in the pre- and post-operative periods. The mean patient body temperature at each time point was similar: pre-operative 36.1 °C (SD 0.5), operative 36.1 °C (SD 0.6) and post-operative 36.3 °C (SD 0.4). We therefore used immediate post-operative temperature to assess the effect of body temperature on blood loss. The patient body temperature was not found to be associated with increased blood loss for any of the three measurement outcomes of blood loss. The results of patient temperature on blood loss are summarized in Table 2, separating each blood loss parameter and temperature group according to surgeon. Although the surgeon was a significant predictor of blood loss outcomes, the interaction with temperature category was not significant. Therefore, overall results were used to assess the relationship between temperature and outcomes.

**Table 2:** Intra-operative and post-operative blood loss by temperature category immediately post-operatively

Outcome		Low temperature group (<36.0 °C) *n* = 26	Low normal temperature group (36.0 °C–36.5 °C) *n* = 51	Normal temperature group (>36.5 °C) *n* = 24	*p* values*
Mean blood volume loss mL (SD)	Surgeon A	218 (184)	155 (269)	137 (199)	0.77, 0.93
	Surgeon B	130 (179)	90 (324)	125 (456)	
	Overall	140 (205)	101 (300)	133 (302)	
Mean haemoglobin loss g/L (SD)	Surgeon A	22.2 (11.8)	26.7 (11.1)	24.1 (6.8)	0.39, 0.53
	Surgeon B	31.1 (10.9)	30.9 (10.0)	25.0 (9.7)	
	Overall	26.4 (12.0)	28.7 (10.7)	24.5 (7.7)	
Mean drain volume mL (SD)	Surgeon A	51 (69)	75 (77)	90 (73)	0.70, 0.48
	Surgeon B	389 (189)	348 (196)	493 (105)	
	Overall	213 (220)	235 (208)	260 (213)	

Surgeon A used intramedullary instrumentation, surgeon B used computer navigation

*p values are for the comparison between (a) low temperature and normal temperature groups and (b) low normal temperature and normal temperature groups

Both univariable and multivariable analyses were used to evaluate factors influencing blood loss in our cohort of TKA patients. The results are outlined in Tables 3 and 4, respectively.

**Table 3:** Univariable analysis of predictors of blood loss following total knee arthroplasty

	Intra-operative blood loss (mL)		Post-operative drain volume (mL)		Change in haemoglobin (g/L)	
Predictors	Regression coefficient (95 % CI)	*p* value	Regression coefficient (95 % CI)	*p* value	Regression coefficient (95 % CI)	*p* value
Age	10.4 (2.3–18.6)	0.01	−3.4 (−8.9 to 2.0)	0.22	0.14 (−0.08 to 0.37)	0.21
Female gender	140.5 (−12 to 293)	0.07	93.6 (−2.1 to 189.3)	0.06	−0.001 (−4.3 to 4.3)	0.99
BMI	3.1 (−13.5 to 19.8)	0.7	9.1 (−1.0 to 19.1)	0.08	−0.38 (−0.83 to 0.07)	0.09
Surgeon	220.3 (71.6 to 369.1)	0.004	317 (253 to 382)	<0.001	−4.90 (−9.08 to −0.72)	0.02
Heart rate	1.35 (−5.3 to 7.95)	0.7	3.9 (−0.7 to 8.5)	0.10	0.02 (−0.17 to 0.22)	0.81
Systolic BP	3.3 (−0.06 to 6.7)	0.054	−1.0 (−3.8 to 1.7)	0.46	0.06 (−0.03 to 0.16)	0.18
Anaesthetic type (reference category is general anaesthetic alone)						
Spinal	−108.57 (−952 to 734.9)	0.80	−393 (−829 to 44)	0.08	−23 (−62 to 16)	0.24
Regional	45 (−921.28– 1011.28)	0.93	154 (−144 to 452)	0.31	−18 (−15 to 52)	0.46
General + regional	−250 (−1161.02 to 661.02)	0.59	−162 (−369 to 46)	0.13	−14 (−34 to 6)	0.17
Spinal + regional	−210 (−0.46 to 568.10)	0.57	−210 (−417 to −4)	0.05	−16 (−36 to 4)	0.12
General + spinal + regional	−166.11 (−965.67 to 633.45)	0.68	−361 (−622 to −99)	0.01	−25 (−47 to −2)	0.03
Operative time	1.19 (−1.36 to 3.74)	0.36	−5.3 (−7.3 to –3.2)	<0.001	0.02 (−0.06 –0.11)	0.61
Medication (reference category is rivaroxaban alone)	NA					
Low-molecular-weight heparin			−312 (−387 to −237)	<0.001	−11 (−22 to 16)	0.06
Warfarin			−205 (−378 to −32.6)	0.02	−10 (−33 to 14)	0.41
Aspirin			−332 (−505 to −159)	<0.001	−6 (−13 to 25)	0.56
Aspirin + Rivaroxaban			97 (−40 to 235)	0.16	17 (−7 to 39)	0.16
Aspirin + LMWH			−287 (−414 to −160)	<0.001	−1 (−22 to 20)	0.92

**Table 4:** Multivariable analysis of predictors of blood loss following total knee arthroplasty

Intra-operative blood loss (mL)		
Predictor	Regression coefficient (95 % CI)	*p* value
Age	10.3 (4.8 to 15.9)	<0.001
Surgeon B	−131 (−235 to −27)	0.01
Drain Volume (mL)		
Predictor	Regression coefficient (95 % CI)	*p* value
Medication (reference category is rivaroxaban alone)		<0.001
Low molecular weight heparin	−282 (−360 to −205)	<0.001
Warfarin	−238 (−406 to −70)	0.01
Aspirin	−300 (−464 to −137)	<0.001
Aspirin + rivaroxaban	133 (−2 to 267)	0.05
Aspirin + LMWH	−232 (−372 to −93)	0.001
Type of anaesthetic (reference category is general anaesthetic alone)		0.02
Spinal	−290 (−621 to 41)	0.09
Regional	24 (−187 to 236)	0.82
General + regional	−193 (−341 to −45)	0.01
Spinal + regional	−201 (−350 to −51)	0.01
General + spinal + regional	−218 (−410 to −26)	0.03
Haemoglobin loss (g/L)		
Predictor	Regression coefficient (95 % CI)	*p* value
Surgeon B	−4.3 (−8.5 to −0.1)	0.04

Using intra-operative volume loss as outcome, predictors of blood loss are increased age and surgical technique. Surgeon B who used computer navigation had on average 131 mL less blood loss than surgeon A. Each year of increased age resulted in 10 mL greater blood loss. Higher pre-operative systolic blood pressure and female gender appeared to increase blood loss but were not statistically significant.

Significant univariable predictors of higher drain volume included surgeon, operative time, type of anaesthesia and type of anticoagulant medication. Using multivariable regression analysis, patients undergoing TKA under general combined with regional anaesthesia, spinal plus regional, or general plus spinal plus regional had significantly less drain volume blood loss compared to general anaesthesia alone. The use of low molecular weight heparin, coumadin, aspirin, or combination of aspirin and low-molecular-weight heparin resulted in significantly less blood loss than the use of rivaroxaban. The use of aspirin in addition to rivaroxaban caused increased blood drainage loss when compared with rivaroxaban alone.

The two variables predictive of total haemoglobin loss are surgeon and requirement for allogenic blood transfusion. Surgeon B had on average 4.9 g/L less haemoglobin loss than surgeon A. The patients who received an allogenic blood transfusion had on average 9.3 g/L more haemoglobin loss.

## Discussion

The results of our study failed to demonstrate any effect of patient temperature during the peri-operative period, within the range we observed between 34.3 to 37.2 °C, on blood loss in primary TKA. Measuring blood loss through intra-operative suction volume, post-operative drainage and post-operative change in haemoglobin, no difference could be demonstrated in patients with temperatures below 36 °C compared to patients with a normal temperature above 36.5 °C. This resulted in no difference in allogenic transfusion rate between the patient temperature groups. Therefore, our hypothesis that maintaining patient temperature at 36.5 °C and above during TKA will result in reduced blood loss was not substantiated.

Our study is not suggesting that anaesthesia-induced hypothermia is inconsequential or that patient temperature monitoring is unimportant. Maintenance of patient temperature may have a greater role in countries where ambient temperatures are usually low. Hypothermia may also have a significant role in other complications such as infection following TKA. What we are suggesting is that within the clinically observed temperature range, using routine patient warming techniques, blood loss was not affected. Therefore, rigid maintenance of patient temperature above 36.5 °C through further active warming techniques may not be necessary in reducing blood loss in TKA.

Our study design does have a number of weaknesses and limitations. The study did not include a control group. We did not feel it would be ethical to intentionally expose patients to a low temperature situation, given what is known from other fields of surgery and current best practice guidelines [17]. We aimed not to determine the effect of active warming, but rather look for a correlation between lower body temperature and blood loss in the primary TKA, which we believe our observational prospective study allowed us to do. Secondly, the patient cohort was derived from two surgeons with differing surgical technique. However, the primary aim of the study was to determine if patient temperature had an effect on blood loss. Both surgeons used the same operating theatres with identical surgical environment. The use of multivariable analysis was able to separate the effects of surgeon technique from other factors to ensure that the study conclusions remain valid. Thirdly, the three cohorts of patient temperature varied by only 0.5–1 °C. Whilst the effect difference is small, we believe this reflects real clinical practice and makes the findings applicable to what surgeons would routinely observe. In addition, previous studies have reported even mild hypothermia (<1^0^ C) significantly increases surgical blood loss by approximately 16 % and the relative risk of transfusion by 22 % [10]. Fourthly, our method for determining intra-operative blood loss may not be the most accurate and reliable approach. We observed a large standard deviation for intra-operative blood loss of 389 mL from a mean of 70 mL and included some patients with a negative difference. The method used does not account for blood on the drapes or blood taken up by surgical sponges. To overcome this weakness, blood loss was also estimated using two other methods including post-operative drain volume and change in haemoglobin. None of the three measures for blood loss in our study were affected by patient temperature, so we feel our conclusions are still valid. Finally, pulmonary artery temperature is considered the gold standard for temperature measurement. Our study used axillary temperature as the best non-invasive method and the most practical technique for daily clinical practice. Axillary temperature has been shown to compare well with pulmonary artery temperature [18] and is equivalent to other invasive measures of core temperature such as intravesical and rectal temperature [19]. Whilst ideally core temperature would have been a more accurate method of measurement, we used a consistent reproducible technique for axillary temperature across the whole patient cohort and therefore feel our methodology is still justifiable and credible. As we recorded temperatures up to 24 h post-operatively, including in the recovery room and surgical ward, using invasive methods was not possible. Our aim was to determine the effect of patient temperature on blood loss even after departure from the operating room.

In our results, there was no correlation between intra-operative blood loss, post-operative drain volume and change in haemoglobin. Hidden blood loss from tissue extravasation, residual knee blood volume and haemolysis commonly observed following TKA is most likely to account for this observed variation [20].

Several other factors were highlighted to affect blood loss during primary TKA in our study. In common with previous literature, our results demonstrated age, surgical technique, anaesthetic type and type of anticoagulant medication used for DVT prophylaxis to impact blood loss. The exact cause of the age effect is not clear and cannot be determined from the data collected in our study. We postulate older patients have higher blood pressures, combined with reduced arterial tone and capillary fragility, causing greater intra-operative blood loss. Although age is a non-modifiable risk factor, it is important to appreciate its impact and to optimize pre-operative haemoglobin more diligently in elderly patients prior to surgery.

There was also a significant difference in blood loss between the two surgeons. A hybrid prosthesis was used by surgeon A and fully cemented prostheses by surgeon B. However blood loss has not been found to be associated with prosthesis type in terms of fixation in TKA [21]. We believe the difference in blood loss relates to instrumentation and type of anticoagulant, with both factors contributing significantly. Surgeon A used intramedullary instrumentation whereas surgeon B used computer-assisted navigation. Computer navigation has previously been demonstrated to produce reduced blood loss [22, 23], as a consequence of avoidance of violating the femoral and tibial intramedullary canals.

Our study reinforces the concept of regional and spinal anaesthesia having a protective effect against blood loss and is the first from our review to show a protective effect in TKA. Regional anaesthesia results in decreased arterial blood pressure [24], whilst spinal anaesthesia leads to venous hypotension and a decreased venous pressure at the surgical site [25].

Rivaroxaban was shown to be associated with a significantly higher drain volume than low-molecular-weight heparin, aspirin and coumadin. Aspirin in combination with rivaroxaban increased drain volume over rivaroxaban alone and resulted in the greatest blood loss. Our study results contrast previous reports showing no significant difference between low-molecular-weight heparin and rivaroxaban and may reflect the surgeon effect on thrombophylactic preference not accounted for by multivariable analysis [26]. The patients continuing coumadin in the peri-operative period with an INR of 2 on the day of surgery was associated with a lower drain volume than patients treated with low-molecular-weight heparin and rivaroxaban. The practice of continuing coumadin without cessation in patients already taking the medication pre-operatively or in patients with previous history of pulmonary emboli was utilized by both surgeons and supports a growing trend that peri-operative bridging therapy and coumadin cessation may not be necessary or the most effective strategy in patients undergoing TKA [27].

## Conclusions

Peri-operative patient temperature, within the clinically observed range from 34.3 to 37.2 °C, did not affect blood loss or the need for allogenic blood transfusion in patients undergoing TKA in our study. Avoiding hypothermia is an important aspect on peri-operative patient care; however, rigid temperature control above 36.50 °C may not be necessary to reduce blood loss in primary TKA and other factors may be more important including anticoagulant and anti-platelet medications use, the use of computer navigation and type of anaesthesia. Routine patient warming techniques that maintain patient temperatures within a reasonable range are sufficient to prevent clinically significant hypothermia-induced blood loss following TKA.
